# Reflex™: a novel method to sequence barcoded long-range PCR products in a pooled population of hundreds of DNA samples

**DOI:** 10.1186/1753-6561-6-S6-P23

**Published:** 2012-10-01

**Authors:** James Casbon, Andrew Slatter, Esther Musgrave-Brown, Robert Osborne, Conrad Lichtenstein, Sydney Brenner

**Affiliations:** 1 Population Genetics Technologies Ltd, Babraham Research Campus, Cambridge CB22 3AT, UK

## 

We are developing novel methods to simultaneously analyze candidate genes or regions from multiple samples in a single experiment rapidly and cost-effectively. One method, we call *Reflex*, provides an elegant approach to sample preparation for long-range PCR (LR-PCR) amplicons. Current methods use LR-PCR for target enrichment then use random fragmentation of each sample followed by ligation of DNA barcodes before sequencing: this approach is expensive and labour intensive.

In the *Reflex* workflow, we perform LR-PCR on genomic targets using primers that also add a DNA barcode to the LR-PCR product. This allows us to then produce pools of the LR-PCR products of multiple, typically 384, samples. The equimolar pooled population of 384 LR-PCR products are then processed to generate tiled 'daughter' *Reflex* PCR products across the target, each carrying a copy of the cognate DNA barcode. This is achieved by an intramolecular fold-back between two inverted *Reflex* sequences, followed by polymerase extension, so copying the DNA barcode. The *Reflex* daughter PCR products are then equimolar pooled for sequencing where the DNA barcode allows identification of the originating DNA sample within the population pool.

We have developed *Reflex* workflows that are compatible with the Illumina, Ion Torrent and Roche-454 next-generation sequencing platforms and can index multiple pooled populations to sequence thousands of DNA samples in a single run. The workflow is robust and can be performed quickly and cheaply with small amounts of input DNA and with high specificity to discriminate between members of multigene families.

